# Association of simple renal cysts and chronic kidney disease with large abdominal aortic aneurysm

**DOI:** 10.1186/s12882-020-01841-6

**Published:** 2020-05-29

**Authors:** Milena Miszczuk, Verena Müller, Christian E. Althoff, Andrea Stroux, Daniela Widhalm, Andy Dobberstein, Andreas Greiner, Helena Kuivaniemi, Irene Hinterseher

**Affiliations:** 1 Vascular Surgery Clinic, Klinik für Gefäßchirurgie, Campus Charité Benjamin Franklin, Hindenburgdamm 30, 12200 Berlin, Germany; 2 Surgical Clinic, Campus Charité Mitte and Campus Virchow-Klinikum, Berlin, Germany; 3 Institute of Radiology, Campus Charité Mitte, Berlin, Germany; 4 Institute of Medical Biometrics and Clinical Epidemiology, Campus Charité Benjamin Franklin, Charité – Universitätsmedizin Berlin, corporate member of Freie Universität Berlin, Humboldt-Universität zu Berlin, and Berlin Institute of Health, Berlin, Germany; 5 Division of Molecular Biology and Human Genetics, Department of Biomedical Sciences, Faculty of Medicine and Health Sciences, Stellenbosch University, Tygerberg, South Africa

**Keywords:** Abdominal aortic aneurysm, renal cyst, chronic kidney disease

## Abstract

**Background:**

Abdominal aortic aneurysms (AAA) primarily affect men over 65 years old who often have many other diseases, with similar risk factors and pathobiological mechanisms to AAA. The aim of this study was to assess the prevalence of simple renal cysts (SRC), chronic kidney disease (CKD), and other kidney diseases (e.g. nephrolithiasis) among patients presenting with AAA.

**Methods:**

Two groups of patients (97 AAA and 100 controls), with and without AAA, from the Surgical Clinic Charité, Berlin, Germany, were selected for the study. The control group consisted of patients who were evaluated for a kidney donation (*n* = 14) and patients who were evaluated for an early detection of a melanoma recurrence (*n* = 86). The AAA and control groups were matched for age and sex. Medical records were analyzed and computed tomography scans were reviewed for the presence of SRC and nephrolithiasis.

**Results:**

SRC (74% vs. 57%; p<0.016) and CKD (30% vs. 8%; p<0.001) were both more common among AAA than control group patients. On multivariate analysis, CKD, but not SRC, showed a strong association with AAA.

**Conclusions:**

Knowledge about pathobiological mechanisms and association between CKD and AAA could provide better diagnostic and therapeutic approaches for these patients.

## Background

Abdominal aortic aneurysm (AAA) is the most common type of aneurysm, and is defined as an abdominal aortic diameter >3 cm [1]. According to recent literature, the prevalence of AAA has decreased in the last decades and is 1–2% [2]. This change can be primarily attributed to a decreased prevalence of smoking [2, 3]. The prevalence of AAA, however, increases with age and is 4.1%–14.2% in men and 0.35 – 6.2% in women > 65 years [4, 5].

As AAA is asymptomatic in the majority of cases [6], it is often initially detected as an incidental finding during ultrasound or computed tomography (CT) examinations. Unfortunately, many AAA cases remain undetected until rupture. The mortality rate of a ruptured AAA is estimated to be 74–90% [7, 8], with a 32–83% pre-hospital mortality rate [7, 9]. One way to reduce this trend, is to implement a national AAA screening program to detect AAA before rupture [10]. Such a program was launched successfully in the USA in 2007 [11] and in the Great Britain in 2009 [12].

A number of risk factors for AAA have been identified. The four primary risk factors are male sex, age > 65 years, smoking and a positive family history [13–18]. Several diseases appear to often co-exist with AAA, including chronic obstructive pulmonary disease (COPD) [13, 19, 20], different types of hernia [20], gallstones [21], and simple renal cysts (SRC) [22]. SRC is a common disease, with increased prevalence in older patients, affecting 24–27% of those > 50 years of age [23, 24]. In older individuals, SCRs are even more common; Carrim et al. found an overall prevalence of 41% [25], while Chang et al. reported a prevalence of 35% in >60-year olds [26].

The co-occurrence of AAA and SRC [22, 27] can be explained by shared risk factors, e.g. older age [26, 28, 29], male sex [25, 30], hypertension [31] and smoking [26]. Ito et al. [22] stated “*the presence of renal cysts shows the strongest independent association with AAA among patients belonging to the 65 to 74 years old group and over 75 years old group*”. The exact pathogenesis of SRC remains unclear, but it is intriguing that both diseases demonstrate increased matrix metalloproteinase (MMP) levels, in the aortic wall in AAA patients [32] and in the cystic fluid in patients with SRCs [33].

Given the potentially shared pathophysiology between SRC and AAA, the primary aim of this study was to assess the prevalence of SRC and other kidney diseases among AAA patients, and compare the results to a group of age- and sex-matched non-AAA patients from the same hospital.

## Methods

The study was approved by the Charité Ethics Committee (approval number: EA1/309/16). Since the study was a retrospective review of medical and imaging records, no informed consent from the patients was required according to the study approval.

### Study groups

This study was a retrospective review of patients’ medical records including radiology records. Two groups of patients were compared in the study. All 197 patients had undergone a computed tomography-angiography (CTA) scan. The first group (n=97) included patients, who underwent AAA surgery in 2004 – 2012 at Charité Clinic Campus Mitte in Berlin, Germany. Surgeries were performed either as elective (unruptured AAA; n=92) or as emergency (ruptured AAA; n= 5) operations. The exclusion criteria were an abdominal aortic diameter <3 cm, AAA operation before 2004, diagnosis of rare genetic disorder such as Marfan syndrome or Ehlers-Danlos syndrome, and the presence of any other arterial aneurysm. Further exclusion criterion was the presence of genetic kidney diseases, such as autosomal dominant polycystic kidney disease (ADPKD). For the AAA patients, the pre-operative scans were used.

The control group (n=100) included patients without AAA investigated at the Institute of Radiology of Charité Clinic, Berlin, Germany, and consisted of patients who were evaluated for a kidney donation in 2005 – 2014 (n = 14) and patients who were evaluated for an early detection of a melanoma recurrence (n = 86). We chose this group of patients as a control group for the following reasons: 1) they were also examined by abdominal CTA; 2) melanoma is an age-related disease and a disease of a different organ, not the aorta; and 3) they were from the same hospital system. Also, there were no differences in the mean height, weight, or BMI between the AAA and control groups [34].

AAA patients and controls were matched on sex and age (± 2 years). For the AAA patients, age at the time of the first AAA diagnosis was used for this analysis. If this information was missing, age at the time of AAA surgery was taken. For the control group, age during the CTA scan was used for the analysis.

### Clinical data

**Fig. 1:**
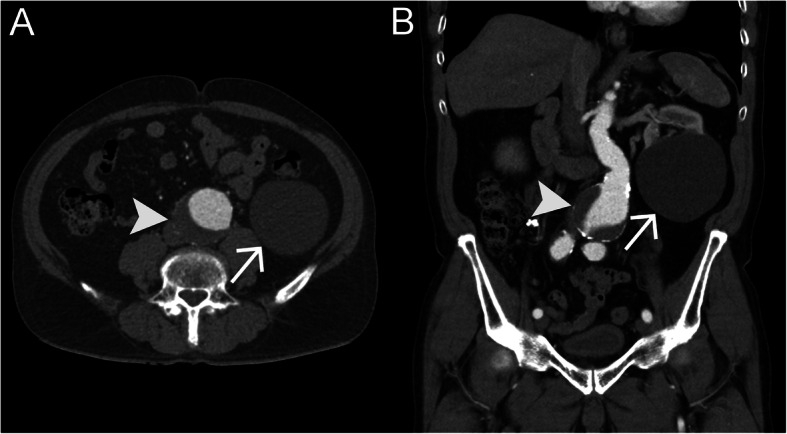
Simple renal cyst detected in a CT scan. Contrast-enhanced CT scan of the abdomen in axial (**a**) and coronal (**b**) plane, arterial phase, demonstrates an AAA (arrowhead) with mural thrombus and patent lumen and a large hypodense mass on the lower left renal pole, representing a simple renal cyst (arrow)

For the analysis of the CTA scans, Centricity eRadCockpit Software (GE Healthcare, Chalfont St Giles, Great Britain) was used. First, written reports from board-certified radiologists were reviewed by one of the authors (M.M.). As SRCs are common, sometimes they were not described as a diagnosis in the report. For that reason, the CTA scans were assessed again for the presence of SRC (Fig 1) and kidney stones. Results were discussed with a board-certified radiologist (C.E.A.).

Individual data on all study patients for all variables used in the study are available in the Additional file 1.

SRC (ICD-10: N28.1) were divided into subgroups according to their size: Group 1: ≤1 cm; Group 2: 1.01 – 3.0 cm; Group 3: 3.1 – 5.0 cm; and Group 4: >5 cm. SRCs were also classified using Bosniak Classification System: I: simple, benign cysts; II: minimally complicated benign cystic lesions; III: more complicated cystic lesions; and IV: cystic carcinoma [35].

Nephrolithiasis (ICD-10: N20.0) was defined as a presence of kidney stones on the CTA scans.

Additional patient data [34] were collected from the medical records using the patient data management program SAP (SAP SE, Walldorf, Germany). Information about the presence of chronic kidney disease (CKD) was collected. CKD was defined as a presence of the following ICD-10 diagnostic codes in the medical records: N18.1 for CKD stage 1, N18.2 for CKD stage 2, N18.3 for CKD stage 3, N18.4 for CKD stage 4, N18.5 for CKD stage 5, and Z94.0 for renal transplantation (Additional file 2). Additional diseases in the same study groups are described in another study [34].

### Statistical analysis

For statistical analyses SPSS Statistics Version 22 for Windows (IBM, Armonk, New York, USA) was used. First, a univariable analysis was carried out. For quantitative variables, the mean, median, standard deviation, minimal and maximal values were determined. The categorical variables were analyzed using cross-tabulation. The differences between the two groups were determined using Mann-Whitney U test or chi-squared test (Fisher’s exact test) where appropriate. A difference was defined significant, if *p*≤0.05.

The univariable analysis was followed by a multivariable analysis to identify independent risk factors. Significant values from the univariable analysis were included in a multiple logistic regression model. This included the following parameters: ever smoker, peripheral artery disease (PAD), pack years of smoking, incisional hernia, any hernia, congestive heart failure, American Society of Anesthesiologists (ASA) score, diabetes mellitus, coronary bypass, creatinine, COPD, current smoker, coronary artery disease, diverticulosis, platelet count [34] and SRC. Parameters with >50% of the values missing were excluded from the analysis. A forward and backward analysis was performed. Odds ratios (OR) and a 95% confidence intervals (CI) were calculated. The analysis was carried out on the entire study group as well as stratified by age (< 65 and ≥ 65 years).

## Results

Our study included 97 (76 male and 21 female) AAA patients and 100 (79 male and 21 female) age- and sex-matched controls. Altogether, 29.9% AAA and 8% control patients had CKD diagnosis in their medical records (*p*<0.001; Table 1). The distribution of CKD stages was also statistically significantly different between the study groups (*p*=0.002; Table 1). One AAA patient received a renal transplantation due to the CKD. Nephrolithiasis was found in 2.1% of the AAA and 7.1% of the control patients (*p*=0.170). SRCs were found amongst 74.2% AAA patients and 57% controls, resulting in a statistically significant difference (Table 1; *p*=0.016).

**Table 1:** Comparison of simple renal cysts and chronic kidney disease between study groups

Variable	AAA group		Control group		*p*^a^
	*With variable* *n*	*Data available* *n*	*With variable* *n*	*Data available* *n*	
CKD, all stages^b^	29	97	8	100	<0.001
CKD stage 1/2/3/4/5/RTX^c^	5/9/11/1/2/1	97	1/4/3/0/0/0	100	0.002
Nephrolithiasis	2	97	7	98	0.170
SRC (both right and left kidney)^d^	72	97	57	100	0.016
SRC, right kidney only	58	97	46	100	0.064
1 – 4 SRC in right kidney^d^	46	97	41	100	0.05
>5 SRC in right kidney^d^	12	97	5	100	0.05
SRC, left kidney only^d^	60	97	33	100	<0.001
1 – 4 SRC in left kidney^d^	48	97	29	100	<0.001
> 5 SRC in left kidney^d^	12	97	4	100	<0.001

^a^Chi-square test

^b^Defined as presence of ICD-10 code in medical records: N18.1 for CKD stage 1, N18.2 for CKD stage 2, N18.3 for CKD stage 3, N18.4 for CKD stage 4, N18.5 for CKD stage 5 or Z94.0 for renal transplantation.

^c^Defined as presence of kidney stones (ICD-10: N20.0) on the CTA scans.

^d^Defined as presence of simple renal cysts (ICD-10: Q61.9) on the CTA scans.

*CKD* Chronic kidney disease; *SRC* Simple renal cyst; *RTX* Renal transplantation.

In the right kidney, SRCs were found in 59.8% of AAA patients and in 46% of controls (*p*=0.064). In the AAA group, 47.4% of patients had 1–4 SRCs, and 12.4% of patients had ≥5 SRCs. In the control group, these numbers were 41% and 5%, respectively (*p*=0.05, for both comparisons; Table 1) . In the right kidney, SRCs ≤1 cm were significantly more common among AAA than control patients (*p*<0.001; Table 2).

**Table 2:** Simple renal cysts in the right kidney classified according to their size

SRC classification based on size	AAA group			Control group			*p*^a^
	*Number of SRC per patient* *Mean+/-SD*	*Median number of SRC per patient*	*Data available* *n*	*Number of SRC per patient* *Mean+/-SD*	*Median number of SRC per patient*	*Data available* *n*	
≤1 cm	1.15 ± 1.86	0	97	0.50 ± 1.45	0	100	<0.001
1.1 cm – 3.0 cm	0.43 ± 0.95	0	97	0.41 ± 0.79	0	100	0.99
3.1 cm – 5.0 cm	0.10 ± 0.34	0	97	0.05 ± 0.26	0	100	0.15
>5 cm	0.02 ± 0.14	0	97	0.04 ± 0.20	0	100	0.68

^a^Mann-Whitney U test

*SRC* Simple renal cyst

In the left kidney, SRCs were found in 62.5% of AAA and 33% of control patients (*p*<0.001; Table 1). In the AAA group, 50% of patients had 1–4 SRCs, and 12.5% of patients had ≥5 SRCs. In the control group, these numbers were 29% and 4%, respectively (*p*<0.001, for both variables; Table 1). In the left kidney, both small (≤1 cm; *p*<0.001) and medium size (1 – 3 cm; p=0.020) SRCs were significantly more common among AAA than control patients (Table 3). In the AAA group, we also found two patients with SRCs classified as Bosniak II and two patients with SCRs classified as Bosniak III. In the control group, one patient each with Bosniak II and Bosniak III were found. There were 46 (47.4%) AAA and 22 (22%) control group patients who had bilateral SRC disease, whereas 25 (25.8%) AAA and 34 (34%) control patients had SRCs in only the left or right kidney (*p*=0.001).

**Table 3:** Simple renal cysts in the left kidney classified according to their size

SRC classification based on size	AAA			Controls			*p*^a^
	*Number of SRC per patient* *Mean+/-SD*	*Median number of SRC per patient*	*Data available,* *n*	*Number of SRC per patient* *Mean+/-SD*	*Median number of SRC per patient*	*Data available,* *n*	
≤1 cm	1.28 ± 2.08	1	96	0.37 ±1.17	0	100	<0.001
1.1 cm – 3.0 cm	0.56 ± 0.95	0	96	0.30 ± 0.73	0	100	0.02
3.1 cm – 5.0 cm	0.10 ± 0.31	0	96	0.04 ± 0.20	0	100	0.10
>5 cm	0.01 ± 0.10	0	96	0.00 ± 0.00	0	100	0.49

^a^Mann-Whitney U test

*SRC* Simple renal cyst

In multivariable analyses we found a strong independent association between AAA and CKD (OR = 5.528; 95%CI = 1.732–17.647; p=0.004). We found no direct association between SRC and AAA (OR = 1.693; 95%CI = 0.615–4.658; *p*=0.308), when adjusting for other variables.

We also carried out the analyses separately for those patients < and ≥ 65 years. In patients < 65 years (29 AAA and 21 control patients), CKD was the only parameter with a statistically higher prevalence in the AAA group (*p* = 0.001; Table 4). In patients ≥ 65 years (68 AAA and 79 control patients), AAA patients suffered significantly more often from CKD (*p* = 0.002), SRC (*p* = 0.018) and SRC on left kidney only (*p* = < 0.001; Table 5). Further, CKD showed an independent association with AAA in multivariate analysis in this older age group (OR = 6.503; 95%CI = 2.088 – 20.255; *p* = 0.001).

**Table 4:** Univariate analysis of AAA in patients < 65 years old

Variable	AAA (*n* = 29)	Controls (*n* = 21)	p
Age (mean ± SD), years	58.7 ±5.7	59.0 ± 4.8	1.000
AAA diameter at first diagnosis (mean ± SD), mm	54.3 ± 19.1	-	-
AAA diameter at surgery (mean ± SD), mm	62.1 ± 16.3	-	-
Male	93.1%	85.7%	0.638
Chronic kidney disease	27.6%	0.0%	0.001
Simple renal cysts	58.6%	38.1%	0.252
Simple renal cysts right kidney	51.7%	28.6%	0.148
Simple renal cysts left kidney	39.3%	23.8%	0.359

Group size not large enough to perform a multivariate analysis.

**Table 5:** Univariate and multivariate analysis of AAA in patients >65 years old

Variable	Univariate analysis			Multivariate analysis		
	AAA (*n* = 68)	Controls (*n* = 79)	p	OR	95%CI	p
Age (mean ± SD), years	73.3 ± 5.8	74.5 ± 6.1	0.169	-	-	-
AAA diameter at first diagnosis (mean ± SD), mm	50.9 ± 13.0	-	-	-	-	-
AAA diameter at surgery (mean ± SD), mm	57.6 ± 10.1	-	-	-	-	-
Male	72.1%	77.2%	0.568	-	-	-
CKD	30.9%	10.1%	0.002	6.503	2.088 – 20.255	0.001
SRC	80.9%	62.0%	0.018	2.5	0.736 – 8.497	0.142
SRC right kidney	63.2%	50.6%	0.136	-	-	-
SRC left kidney	72.1%	35.4%	< 0.001	-	-	-

## Discussion

The main findings of our study were that CKD was more frequent in AAA than age- and sex-matched control patients and showed a strong association with AAA in multivariable analysis, which included ever smoker, PAD, pack years, incisional hernia, any hernia, congestive heart failure, ASA score, diabetes mellitus, coronary bypass, creatinine, COPD, current smoker, coronary artery disease, diverticulosis, platelet count and SRC. AAA patients also had a higher rate of SRC, but SRCs were not independently associated with AAA.

Our study demonstrated a strong association between AAA and CKD with 29.9% of the AAA and only 8% of the age- and sex-matched control patients diagnosed with CKD. The AAA patients also exhibited a more advanced CKD stage. Previously published studies reported a wide range of CKD prevalence (3–65%) among AAA patients [20, 36–39]. Alnassar et al. [36] and Pitoulias et al. [20] found no significant difference in the prevalence of CKD between AAA and PAD patients [20, 36]. However, patients with a large AAA (>5.5 cm) had a significantly higher rate of CKD than patients with a small AAA (13% vs. 2%) [20]. Approximately half (52.6%) of the AAA patients in our study had a large AAA (>5.5 cm) and all were operated on for AAA, and the rate of CKD was over four times as high as the rate in the study of Pitoulias et al [20]. Furthermore, similar to the findings by Chun et al. [38] and Takeuchi et al. [39], our study demonstrated an independent association of AAA and CKD in multivariable analysis, not seen in the study by Pitoulias et al. [20].

We estimated the prevalence of SRC to be 74.2% in the AAA and 57% in the control group. Based on previous literature, the SRC prevalence in general population varies 4.2–41% [25, 28], which is lower than in the current study (Table 6). Similarly, the SRC prevalence among AAA patients in the current study was higher than in most of the previous studies, which reported a prevalence of 38–69% among AAA patients and 19–45% for controls [20, 22, 27, 40–42]. A recent study by Brownstein et al. [42] analyzed a total of 35,498 patients who underwent both chest and abdominal CT imaging during a 4-year period. Altogether 18% of these patients had SRC and 2.6% had AAA. Compared with the matched population without SRC, patients with SRC demonstrated an increased prevalence of AAA (8% vs. 3%). They were also more likely to have thoracic, ascending and descending aortic aneurysms or dissections [42]. Five previous studies found an independent association between AAA and SRC in a multivariable analysis [20, 22, 27, 40, 42], but we could not confirm this in our study. However, three of those studies [22, 40, 42] examined a significantly larger patient group.

**Table 6:** Prevalence of SRC in AAA and control patients in published studies

Study reference and characteristics of the study		Prevalence of SRC (%)		Univariate analysis		Multivariate analysis	
	Number of AAA/controls	AAA	Controls	*p* value	OR (95%CI)	*p* value	OR (95%CI)
Current study	97/100	74.2	57	0.016	NA	0.30	1.69 (0.62 – 4.66)
Pitoulias et al. [20] • AAA vs. aortoiliac occlusive disease • SRC diagnosed on CT	110/60	69	27	<0.001	0.16 (0.08 – 0.33)	<0.001	0.23 (0.11 – 0.48)
Ito et al. [22] • Retrospective study • AAA vs. patients who underwent CT for preoperative evaluation of thoracic and cardiovascular surgery • Exclusion criteria: hemodialysis • SRC diagnosed on CT	16/102^a^ 56/88^b^ 52/81^c^	38^a^ 63^b^ 56^c^	29^a^ 37^b^ 38^c^	0.56^a^ 0.002^b^ 0.05^c^	NA	0.002^b^ 0.02^c^	4.15^b^ (1.72 – 10.03) 3.00^c^ (1.16 – 7.73)
Song et al .[40] • Retrospective study • AAA vs. patients who underwent CT as part of a health screening program • Exclusion criteria: ruptured AAA, end-stage renal disease (dialysis, transplantation), history or family history of genetic cystic diseases (tuberous sclerosis, von Hippel-Lindau disease, and ADPKD), hydronephrosis, complex cysts, solid masses, peripelvic cysts • SRC diagnosed on CT	271/1,387	55^d^ 56^e^	19^d^ 29^e^	0.001^d^ 0.03^e^	NA	0.04	2.64 (1.05 – 6.63)
Spanos et al. [41] • Retrospective study • Only male patients, age-matched study groups • AAA vs. patients who underwent CT for other reasons • Exclusion criteria: ADPKD • SRC diagnosed on CT	100/100	63	45	NA	NA	0.02^f^	NA
Yaghoubian et al. [27] • Retrospective study • Age and sex-matched study groups • AAA vs. patients who underwent CT for traumatic injury • SRC diagnosed on CT	100/100	54	30	0.0006	2.73 (1.53 – 4.9)	0.03^f^	2.05^f^ (1.08 – 3.88)

^a^Patients <65 years.

^b^Patients 65 – 74 years.

^c^Patients >75 years.

^d^Data for the entire sample group.

^e^Data for the matched groups.

^f^AAA as predictive factor for SRC.

*NA* No data available; *CI* Confidence interval; *OR* Odds ratio; *SRC* Simple renal cyst.

Ito et al. [22] found an association between AAA and SRC in their multivariable analysis, but only in patients > 65 years. Because of low patient numbers, we were not able to analyze the patients < 65 years separately. Our analyses with the older patients (≥ 65 years) showed a significantly higher prevalence of CKD in the AAA group (31% vs. 10%) with an independent association in the multivariate analysis (OR = 6.5, *p* = 0.001). Also, SRC were significantly more frequent in the AAA group (81% vs. 62%, *p* = 0.018), however, we were not able to confirm an independent association in the multivariate analysis. Therefore, our analysis partially confirm the results previously published by Ito et al. [22]. Age is also an important risk factor for SRC. Prior studies have confirmed that SRC develops mostly at older age [24, 25, 28, 29, 40, 41], and Ito et al. [22] and Yaghoubian et al. [27] found a significant difference in the age of patients with and without SRC in the general population.

SRC and AAA share some common risk factors, e.g. older age, male sex and hypertension [25, 26, 28–31], and some studies also mention smoking as a possible risk factor for SRC [26]. Molecular studies suggest that MMPs play a role in the pathophysiology of SRC and AAA [33]. Furthermore, one study on 108 autopsies showed a correlation between the diameter of the abdominal aorta and the number of SRC [43].

Further research on kidney diseases and AAA is not only of academic, but also of clinical interest. Nowadays, the majority of AAA are repaired using endovascular aneurysm repair (EVAR), which requires a contrast agent administration, known to be nephrotoxic. Only one study has investigated the kidney function in patients with SRC after EVAR [41], and found that patients with SRC had slightly higher creatinine levels, both before and after surgery, but the difference was not statistically significant [41]. There was no significant difference in the creatinine levels after EVAR [41], leading to the conclusion that kidney function is not affected by the presence of SRC. As we have reported previously, in the current study population, the AAA group patients had significantly higher creatinine levels than the control group patients [34].

Nephrolithiasis is a common problem in the elderly population. A higher prevalence of nephrolithiasis has been reported in patients with SRC [26] and ADPKD [44]. The relationship between renal stones and AAA has not been investigated previously. As SRC appear frequently in AAA patients [22, 27, 45], one might expect that nephrolithiasis affects AAA patients as well. In our study population, we found no association between AAA and nephrolithiasis. Nonetheless, when examining a patient with a renal colic, one should consider a symptomatic or ruptured AAA as a potential differential diagnosis.

The association between AAA and CKD also requires further research by examining the role CKD plays on the development and progression of AAA. It has been reported that blood vessel walls in patients with CKD are thinner, which can increase the risk for rupture [46]. The co-occurrence of AAA and CKD also has several clinical implications, since CKD increases the rate of complications after surgery [47]. The clot inside the aneurysm sac can also impair the blood perfusion of renal arteries (e.g. by embolization).

Further, CKD patients are known to suffer from vitamin D deficiency [48]. Wong et al. [49] reported that vitamin D deficiency may be a potential risk factor for AAA, but the association was weak with OR = 1.23 (95%CI = 0.87-1.73). Thus, the literature on the association between AAA and vitamin D levels is still sparse and additional studies are required.

The main limitation of our study is the fact that this was a retrospective single-center study with a small number of patients and controls. Another possible limitation is the fact that the control group consisted of patients with melanoma and those evaluated for kidney donation and their comorbidity profile might not be representative of the general population. Especially patients with melanoma may have had a stronger sun exposure and therefore could have higher vitamin D levels which are potentially associated with AAA and CKD. However, a comparison of our two control groups (kidney donation vs. melanoma) showed no difference in the prevalence of CKD or SRC between these two groups (both *p* > 0.05, not shown). Therefore, we assume that the potential bias caused by the vitamin D levels in melanoma patients is of minimal importance.

Our study evaluated patients with larger AAAs which were treated surgically. This might have caused a selection bias and the results might not be representative of all AAA patients. A major advantage of our study was matching of patients and controls on sex and age minimizing the confounding effects attributed to these factors.

## Conclusions

A better understanding about the pathophysiology of AAA will facilitate the development of pharmacotherapies for AAA. Also, this knowledge could be used for a better risk stratification. By introducing a national screening program in every country, AAA could be detected earlier. It is also important to consider the possible complications arising from CKD, both after open aneurysm repair, and EVAR. This group of patients should be given special attention and risk factor analysis should be carried out. The risk of rupture should be high enough to justify the risk of surgery. Further research is also needed on patients with small AAA who develop problems in kidney function. It remains to be determined if renal function is also affected by an expansion and different types of AAA where the renal arteries are involved.

## Supplementary information

**Additional file 1.** Dataset containing all information on every patient included in the study (Excel format).

**Additional file 2: Table S1.** Data dictionary, in alphabetical order. Includes detailed information about collected data. **Table S2.** Comparison of height, weight, and BMI between the AAA and control group. **Table S3.** SA-Scores. **Table S4.** AB0-blood groups. **Table S5.** Rhesus factor.

## Data Availability

All relevant data are within the paper and the files part of the Supporting Information.

## Abbreviations

AAA: Abdominal aortic aneurysm
ADPKD: Autosomal dominant polycystic kidney disease
ASA: American Society of Anesthesiologists
CI: Confidence interval
CKD: Chronic kidney disease
COPD: Chronic obstructive pulmonary disease
CT: Computed tomography
CTA: Computed tomography angiography
EVAR: endovascular aneurysm repair
OR: Odds ratio
PAD: Peripheral artery disease
SRC: Simple renal cyst
