# Supplementary material for: Association of simple renal cysts and chronic kidney disease with large abdominal aortic aneurysm
Source: BMC Nephrol. 2020 May 29;21:201. doi: 10.1186/s12882-020-01841-6 (PMC7257207; doi:10.1186/s12882-020-01841-6)
Supplement: Supplementary file 2 — Additional file 2: Table S1. Data dictionary, in alphabetical order. Includes detailed information about collected data. Table S2. Comparison of height, weight, and BMI between the AAA and control group. Table S3. SA-Scores. Table S4. AB0-blood groups. Table S5. Rhesus factor. [file 12882_2020_1841_MOESM2_ESM.pdf]

SUPPLEMENTARY MATERIAL

Miszczuk et al. Association of simple renal cysts and chronic kidney disease with large abdominal aortic aneurysm

Supplemental Table I: Data dictionary, in alphabetical order

**Parameter**	**Definition**	**Obtained from**
AAA diameter	At the time of initial diagnosis and surgery, in mm	CT
Age	AAA group: Age at initial diagnosis or, if missing, surgery<br>Control group: Age at CT scan	Medical records
Artificial pacemaker	ICD-10: Z95.0	Medical records
ASA score	• ASA I – Normal, healthy patient<br>• ASA II – Patient with mild systemic disease<br>• ASA III – Patient with severe systemic disease<br>• ASA IV – Patient with severe systemic disease that is a constant threat to life<br>• ASA V – Moribund patient who is not expected to survive without the operation<br>• ASA VI – Declared brain-dead patient whose organs are being removed for donor purposes	Medical records
Atrial fibrillation	ICD-10: I48.9	Medical records
Blood group	A/B/AB/0	Medical records
BMI	In kg/cm^2	Medical records
Cholecystolithiasis	• Cholecystolithiasis ICD-10: K80.2<br>• Cholecystectomy ICD-10: Z90.4	CT
Chronic kidney disease	Chronic kidney disease:<br>• Stage 1 ICD-10: N18.1<br>• Stage 2 ICD-10: N18.2<br>• Stage 3 ICD-10: N18.3<br>• Stage 4 ICD-10: N18.4<br>• Stage 5 ICD-10: N18.5	Medical records
Congestive heart failure	ICD-10: I50.0<br>NYHA class I/II/III/IV	Medical records
Connective tissue disease	• Marfan syndrome ICD-10: Q87.4<br>• Ehlers-Danlos syndrome ICD-10: Q79.6<br>• Loeys-Dietz syndrome ICD-10: Q25.4	Medical records
COPD	ICD-10: J44.90-93	Medical records

Coronary artery disease	ICD-10: I25.1	Medical records
Coronary bypass	ICD-10: Z95.1	Medical records
Diabetes	ICD-10: E11.90<br>Treatment with:<br>• Diet<br>• Medication<br>• Insulin	Medical records
Diverticulosis	• Diverticulosis ICD-10: K57.0<br>• Diverticulitis ICD-10: K57.12<br>• Sigma resection ICD-10: Z90.4	CT
Ejection fraction	In %	Medical records
Family history of AAA	Does patient have any first or second degree relatives with AAA	Medical records
Height	In centimeters	Medical records
Hernia	Present or status post-surgery<br>• Inguinal hernia ICD-10: K40.0<br>• Umbilical hernia ICD-10: K42.0	CT
Hyperlipidemia	ICD-10: E78.9	Medical records
Hiatal hernia	ICD-10: K44.9	CT
Hypertension	ICD-10: I10.00 or medication	Medical records
Hypothyroidism	ICD-10: E03.9	Medical records
Liver cysts	ICD-10: K76.8<br>Size<br>• ≤ 1 cm<br>• > 1 – ≤3 cm<br>• > 3 – ≤5 cm<br>• > 5 cm	CT
Lung function (FEV1/VC)	In %	Medical records
Myocardial infarction	ICD-10: I25.2	Medical records
Nephrolithiasis	ICD-10: N20.0	CT
Other aneurysm	Presence of aneurysms in other arteries	CT
Pancreatic cysts	ICD-10: K86.2	CT
Peripheral artery disease	ICD-10: I70.29<br>• Fontaine Stage I/IIa/IIb/III/IV<br>• Bypass ICD-10: Z95.88<br>• PTA ICD-10: Z95.88	Medical records
PTCA	ICD-10: Z95.88	Medical records
Renal cysts	ICD-10: N28.1<br>Size<br>• ≤ 1 cm<br>• > 1 – ≤3 cm<br>• > 3 – ≤5 cm	CT

	• > 5 cm	
Rhesus factor	Rh +/Rh -	Medical records
Smoking	• Current or former smoking ICD-10: F17.2<br>• Years and amount of smoking (pack years)<br>• Years since stopped smoking	Medical records
Splenic cysts	ICD-10: D73.4	CT
Stroke	• TIA ICD-10: G45.99<br>• Stroke ICD-10: I64	Medical records
Surgical material used	Graft material used in AAA surgery	Medical records
Urgency of AAA surgery	Elective, urgent, emergent	Medical records
Weight	In kilograms	Medical records

AAA, abdominal aortic aneurysm; ASA score, American Society of Anesthesiologists; BMI, body mass index; COPD, chronic obstructive pulmonary disease; CT,computer tomography; FEV1/VC, Tiffeneau-Pinelli index, lungfunction; ICD-10, International Classification of Diseases; NYHA, New York Heart Association; PTA, percutaneous transluminal angioplasty; PTCA, percutaneous transluminal coronary angioplasty; TIA, transient ischemic attack.

Supplemental Table II: Comparison of height, weight, and BMI between the AAA and control group

**Variable**	**AAA (n = 96)**		**Control (n = 98)**		**P value [a]**
	Mean+/-SD	Median	Mean+/-SD	Median	
Height, cm	173.29 ± 8.78	174	174.01 ± 8.05	175	0.569
Weight, kg	80.37 ± 15.82	80	79.48 ± 13.99	76.50	0.383
BMI, kg/m^2	26.72 ± 4.7	26.56	26.18 ± 3.66	25.59	0.307

[a] Mann-Whitney U test

Data were not available on 1 AAA patient and 2 controls.

Supplemental Table III: ASA-Scores

Study group	ASA I	ASA II	ASA III	ASA IV	ASA V	Data available on (n)	Average ASA-Score
AAA group	1	21	70	3	2	97	2.84 ± 0.589
Control group	3	34	61	1	1	100	2.63 ± 0.614

Supplemental Table IV: AB0-blood groups

Study group	A	B	AB	0	Data available on (n)	P value Chi-square-test
AAA group	40	15	6	34	95	0.922
Control group	31	12	3	23	69	

Supplemental Table V: Rhesus factor

Study group	Positive, n	Negative, n	Data available on (n)	P value Chi-square-test
AAA group	80	15	95	0.833
Control group	57	12	69	
